# Hypomethylation and up-regulation of *PD-1* in T cells by azacytidine in MDS/AML patients: A rationale for combined targeting of PD-1 and DNA methylation

**DOI:** 10.18632/oncotarget.3324

**Published:** 2015-03-18

**Authors:** Andreas D. Ørskov, Marianne B. Treppendahl, Anni Skovbo, Mette S. Holm, Lone S. Friis, Marianne Hokland, Kirsten Grønbæk

**Affiliations:** ^1^ Department of Hematology, Rigshospitalet, Copenhagen University Hospital, Copenhagen, Denmark; ^2^ Department of Biomedicine, Aarhus University, Aarhus, Denmark; ^3^ FACS Core Facility, Aarhus University, Aarhus, Denmark; ^4^ Department of Hematology, Aarhus University Hospital, Aarhus, Denmark; ^5^ Department of Hematology, Odense University Hospital, Odense, Denmark

**Keywords:** myelodysplastic syndromes, hypomethylating agents, DNA methylation, programmed death-1, T cells

## Abstract

The hypomethylating agents (HMAs) are standard therapy for patients with higher-risk myelodysplastic syndrome (MDS); however, the majority of the patients will lose their response to HMAs over time due to unknown mechanisms. It has recently been shown that T cell expression of the immunoinhibitory receptor PD-1 is regulated by DNA methylation. In 12 of 27 patients (44%) *PD-1* promoter demethylation was observed in sorted peripheral blood T cells isolated over consecutive cycles of treatment with 5-azacytidine (5-aza). The *PD-1* promoter demethylation correlated with an increase in PD-1 expression. Moreover, demethylation of the *PD-1* promoter correlated with a significantly worse overall response rate (8% vs. 60%, *p* = 0.014), and a trend towards a shorter overall survival (*p* = 0.11) was observed. A significantly higher baseline methylation level of the *PD-1* promoter was observed in T cells of non-responding patients compared to healthy controls (*p* = 0.023).

Accordingly, in addition to their beneficial function, HMAs induce PD-1 expression on T cells in the MDS microenvironment, thereby likely hampering the immune response against the MDS blasts. Thus, we suggest that activation of the PD-1 checkpoint during HMA treatment can be a possible resistance mechanism, which may be overcome by combination therapy with a PD-1 pathway inhibitor.

## INTRODUCTION

Myelodysplastic syndromes (MDS) are clonal hematopoietic stem cell disorders, characterized by increased proliferation and aberrant differentiation combined with a high rate of apoptosis [1]. This results in ineffective hematopoiesis and peripheral blood cytopenias as well as an increased risk of developing acute myeloid leukemia (AML). Compared to conventional care regimens, hypomethylating agents (HMAs) have resulted in improved outcomes in MDS [2–4], including delayed leukemic transformation [4] and prolonged survival in patients with higher-risk MDS [2]. 5-azacytidine (5-aza) treatment has also prolonged overall survival (OS) in patients with AML with 20–30% bone marrow blasts [5], and decitabine (5-aza-2′deoxycytidine) has improved the response rates in older patients (> 65 yrs) with newly diagnosed AML [6]. In addition, HMAs are also approved by the U.S Food and Drug Administration and the European Medicines Agency for treatment of chronic myelomonocytic leukemia (CMML). Still, only about 50% of the HMA treated patients achieve a clinical response, the majority will lose response over time [2], and the outcome after HMA failure is poor with a median survival of only 5.6 months [7]. Consequently, there is an urgent need for efficient new treatment modalities in MDS.

The molecular mechanisms of action of HMAs are currently not completely understood. Reactivation of silenced tumor suppressor genes has been suggested to be a key event [8]; however, others and we have also shown that HMAs induce the expression of tumor antigens such as cancer testis antigens (CTAs) on the malignant cells [9, 10]. Since CTAs are normally only expressed at immune privileged sites, e.g. in testicular germ cells, expression of CTAs by the MDS blasts can stimulate an anti-tumor immune response by induction of effector T cells of the adaptive immune system [11–13]. By contrast, the mechanisms of resistance to HMAs are largely unknown.

The programmed death-1 (PD-1) is an immunoinhibitory receptor mainly expressed on activated T cells [14]. Two ligands for PD-1 are currently known, PD-L1 (B7-H1) and PD-L2 (B7-DC). Under normal circumstances both ligands are expressed in low levels in a wide variety of cell types, but surface expression of the protein is rare [15]. The major role of PD-1 is to limit T cell effector responses in peripheral tissues in relation to infection and inflammation, and to limit autoimmunity [14, 16]. In addition, the PD-1/PD-L1 pathway plays an important role in tumor immune evasion and growth [17]. Interestingly, it was recently shown that expression of PD-1 on T cells is regulated by DNA methylation [18]. Hypomethylation of the *PD-1* promoter was observed in CD8^+^ T cells with inhibited function, referred to as exhausted T cells. Interestingly, *in vitro* studies showed that treatment with an HMA increases the expression of PD-1 on activated T cells [18].

PD-L1 and, to a lesser extent, PD-L2 are overexpressed in various types of human tumors, including hematological malignancies such as MDS and AML [19–23]. An increasing amount of data indicate that interactions between PD-1 and its ligands are important mechanisms of immune suppression in the tumor microenvironment [15, 19, 20].

The objective of this study was to investigate the *in vivo* effect of HMA on *PD-1* methylation and expression in T cells obtained from patients during 5-aza treatment, and to evaluate the rationale of combining HMA with a PD-1 pathway inhibitor in MDS.

## RESULTS

### PD-1 methylation in healthy individuals

**Figure 1:**
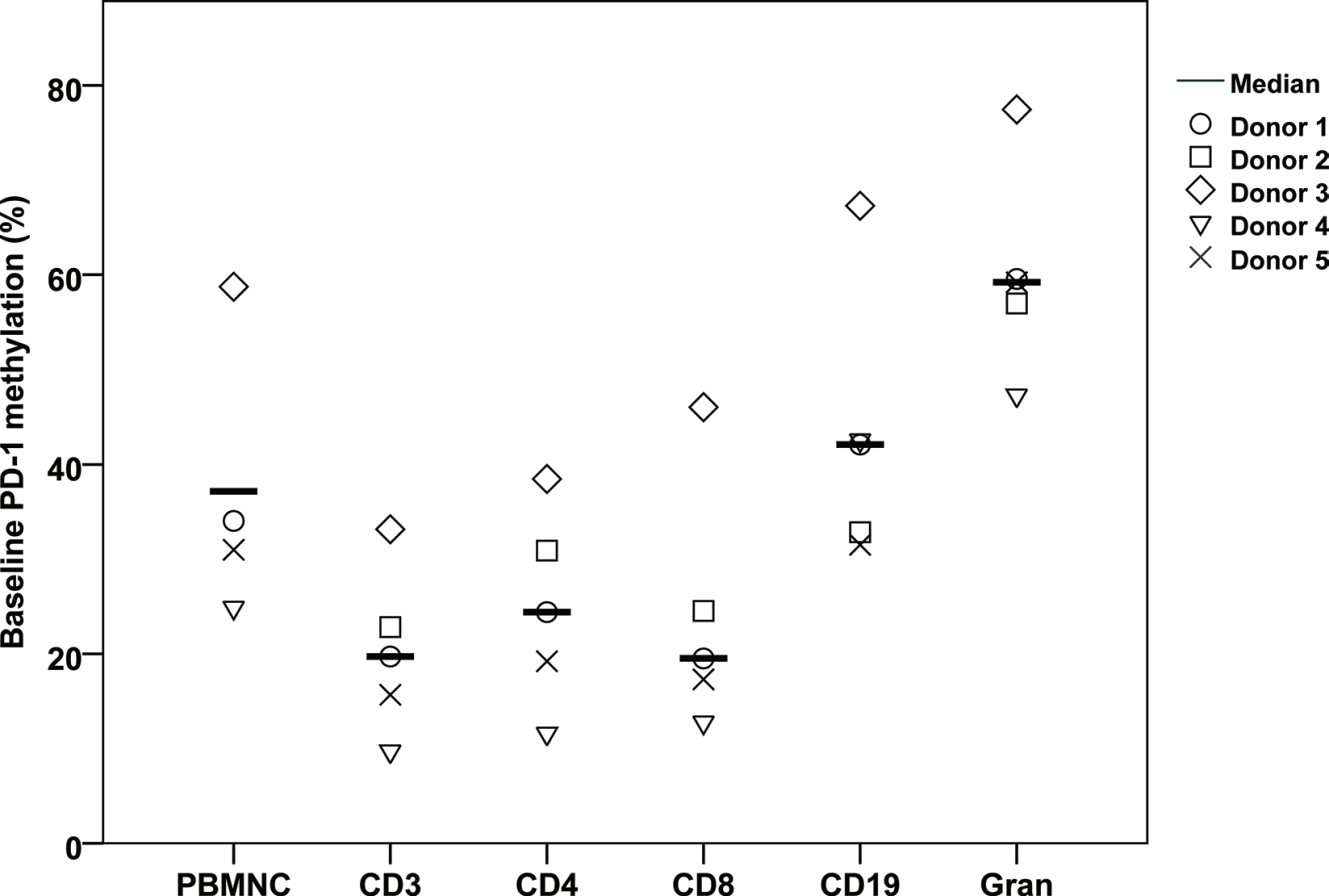
Mean *PD-1* promoter methylation in six distinct cell populations from 5 healthy donors All cells are from peripheral blood. The graph shows the means. PBMNC = peripheral blood mononuclear cells. CD3 = CD3^+^ T cells. CD4 = CD4^+^ T cells. CD8 = CD8^+^ T cells. Gran = granulocytes. B cells = CD19^+^ B cells.

First, we evaluated the level of *PD-1* promoter methylation in peripheral blood mononuclear cells (PBMNCs), granulocytes, CD3^+^ T cells, CD4^+^ T cells, CD8^+^ T cells, and CD19^+^ B cells from five healthy donors (Figure 1). The mean *PD-1* methylation level was: PBMNCs 37.2% (range 24.9–58.7), granulocytes 60.1% (range 47.3–77.5), CD3^+^ T cells 20.2% (range, 9.7–33.2), CD4^+^ T cells 24.9% (range 11.6–38.5), CD8^+^ T cells 24.0% (range 12.8–46.0) and CD19^+^ B cells 43.3% (range 31.5–67.3). The analyses revealed a varying methylation level both among the different cell types and donors. The T cell population carried the lowest level of *PD-1* promoter methylation, which is in line with the fact that the highest *PD-1* gene expression is observed in T cells.

### PD-1 methylation in peripheral blood mononuclear cells from 5-aza treated patients

**Figure 2:**
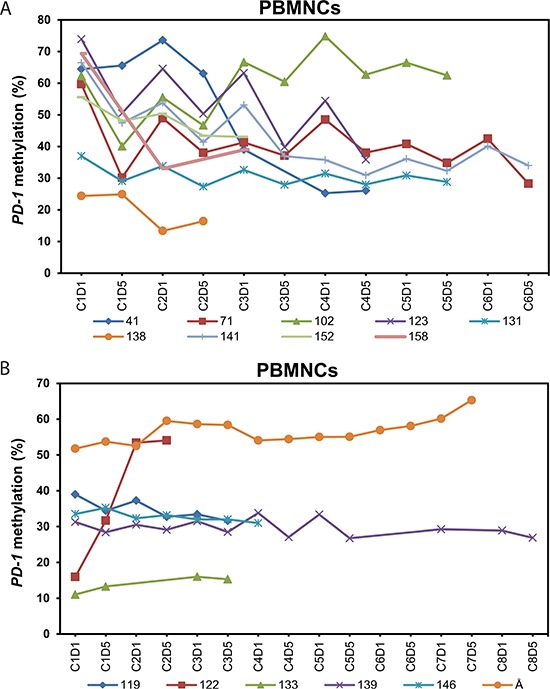
Dynamics of *PD-1* promoter methylation in peripheral blood mononuclear cells of 15 patients during treatment with 5-azacytidine **(A)** *PD-1* promoter methylation in the 9 patients in whom we observed a classifiable demethylation. **(B)** *PD-1* promoter methylation in the 6 patients in whom we did not observe a classifiable demethylation. C = course of 5-aza treatment. D = day in treatment course. PBMNC = peripheral blood mononuclear cells.

Next, we investigated the status of *PD-1* promoter methylation in PBMNCs sampled from patients during the course of 5-aza treatment. Initially, we analyzed unsorted PBMNCs from 15 (12 MDS, 1 AML and 2 CMML) 5-aza treated patients (patient characteristics, see Table 1). Samples from day one and day five of each treatment cycle were analyzed. The patients had received a median number of four cycles of 5-aza (range 2–13). A total of 121 peripheral blood (PB) samples were analyzed. Nine of 15 (60%) patients demonstrated a significant decrease in *PD-1* promoter methylation after 5-aza administration compared to the pre-treatment level (Figure 2A). Demethylation was defined relative to the baseline methylation level based on the following criteria: A statistically significant decrease in methylation level and a decrease of ≥ 10%-points. The demethylation should furthermore occur in ≥ 2 distinct treatment cycles.

**Table 1:** Patient characteristics RA = refractory anemia. RCMD = refractory cytopenias with multilineage dysplasia. RAEB = refractory anemia with excess blasts. AML = acute myeloid leukemia. CMML = chronic myelomonocytic leukemia. IPSS = international prognostic scoring system. IWG = International Working Group. CR = complete remission. 5-aza = 5-azacytidine.

	All	*PD-1* demethylation	No *PD-1* demethylation	*p* value
**N**	27	12 (44%)	15 (56%)	
**Age** median (range)	65 (46–82)	67 (52–82)	66 (46–77)	.79
**Sex**				
Male	14	6	8	.86
Female	13	6	7	
**WHO diagnosis**				
RA	2	0	2	.49
RCMD	3	1	2	1
RAEB-1	2	0	2	.49
RAEB-2	12	6	6	.60
AML	5	4	1	.14
CMML-2	3	1	2	1
**IPSS**				
Low	0	0	0	-
Int-1	7	1	6	.091
Int-2	9	6	3	.13
High	9	4	5	1
AML (>30% blasts)	1	1	0	-
Missing	1	0	1	-
**Cytogenetics (IPSS)**				
Good	8	2	6	.24
Intermediate	10	4	6	1
Poor	9	6	3	.11
**Prior treatment**	16	6	10	.45
Chemotherapy	8	6	5	
Growth factor treatment	8	1	7	
No prior treatment	11	6	5	
**Time (months) from diagnosis until 5-aza-start** median (range)	4 (0–37)	6.5 (0–11)	3 (0–37)	.83
**Median no. of cycles of 5-aza**	5 (2–14)	4 (2–13)	5 (3–14)	.40
IWG 2006 Response				
CR	3 (11%)	1 (8%)	2 (13%)	1
Overall response	10 (37%)	1 (8%)	9 (60%)	.014
**Overall survival** (days) (median)	309	216	334	.11 (log rank)

We observed that the baseline level as well as the continuous level of *PD-1* promoter methylation varied among the patients during 5-aza treatment with different methylation patterns over time. In nine patients *PD-1* promoter demethylation was observed, mostly followed by a diverse remethylation just before the start of the next cycle. In the remaining six patients a stable methylation level, or a slight gain in methylation, was seen throughout the entire treatment period (Figure 2B). The mean baseline methylation level was significantly higher in the group of patients in whom we observed a demethylation of the *PD-1* promoter, 57.0% (SD, 16.2) vs. 30.4% (SD, 15.0), *p* = 0.007, 95%-CI [8.7;44.5]. Taken together, these results reveal that 5-aza does demethylate the *PD-1* promoter in patients PBMNCs *in vivo*.

### PD-1 promoter methylation in CD4^+^ and CD8^+^ T cells from 5-aza treated patients

Since the observed changes in methylation level in PBMNCs could either be a result of a real change in methylation or simply a result of an alteration in the composition of the mononuclear cell subpopulations, we extended our analyses to include isolated T lymphocyte subsets. In 22 (15 MDS, 4 AML, 3 CMML, patient characteristics see Table 1) of the 27 patients we investigated the *PD-1* promoter methylation in sorted CD4^+^ and CD8^+^ T cells. *PD-1* promoter methylation was examined in both CD8^+^ T cells and CD4^+^ T cells because of their cooperative function in tumor surveillance. Ten of the 22 patients were also included in the analyses of PBMNCs described above.

**Figure 3:**
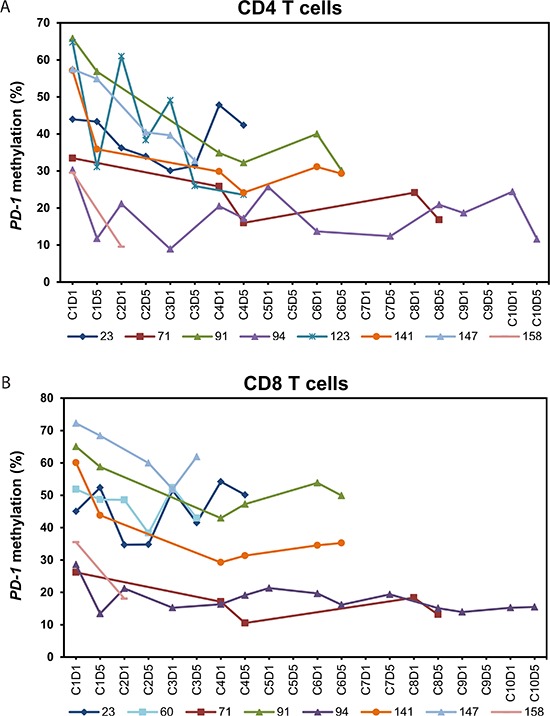
Dynamics of *PD-1* promoter methylation in peripheral blood CD4^+^ and CD8^+^ T cells from the patients with *PD-1* promoter demethylation during treatment with 5-azacytidine **(A)** *PD-1* promoter methylation in CD4^+^ T cells of eight patients. **(B)** *PD-1* promoter methylation in CD8^+^ T cells of eight patients. In patient no. 123 we only observed demethylation in the CD4^+^ T cells (CD8^+^ T cells from patient no. 123 are included in Figure 4B) and in patient no. 60 we only observed demethylation in the CD8^+^ T cells (CD4^+^ T cells from patient no. 60 are included in Figure 4A). C = course of 5-aza treatment. D = day in treatment course.

**Figure 4:**
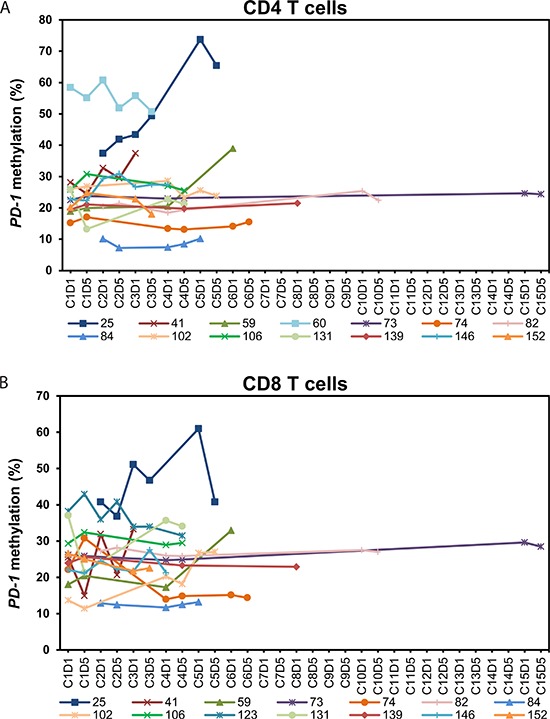
Dynamics of *PD-1* promoter methylation in peripheral blood CD4^+^ and CD8^+^ T cells from patients without *PD-1* promoter demethylation during treatment with 5-azacytidine **(A)** *PD-1* promoter methylation in CD4^+^ T cells of 14 patients. **(B)** *PD-1* promoter methylation in CD8^+^ T cells of 14 patients. In patient no. 123 we observed demethylation in the CD4^+^ T cells (CD4^+^ T cells from patient no. 123 are included in Figure 3A) and in patient no. 60 we observed demethylation in the CD8^+^ T cells (CD8^+^ T cells from patient no. 60 are included in Figure 3A). C = course of 5-aza treatment. D = day in treatment course.

**Figure 5:**
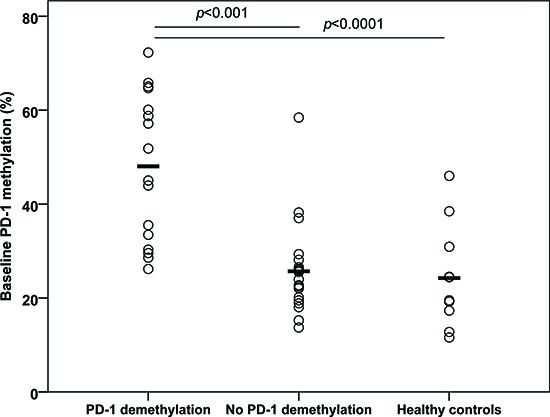
Baseline *PD-1* promoter methylation in peripheral blood CD4^+^ and CD8^+^ T cells from 5-azacytidine treated patients and five healthy donors The patients are grouped according to whether the *PD-1* promoter demethylates or not during treatment. The values for CD4^+^ and CD8^−^ T cells are pooled.

The 22 patients had received a median number of five courses of 5-aza (range 3–14). A total of 132 PB samples were sorted and analyzed. Nine (41%) of the patients demonstrated a significant decrease in *PD-1* promoter methylation in the T cell compartment after 5-aza administration (Figure 3A and 3B). In two of these patients we only observed demethylation in either the CD4^+^ T cells or the CD8^+^ T cells. In the remaining 13 patients an increase in *PD-1* methylation or no methylation changes were observed (Figure 4A and 4B). The mean baseline methylation level in T cells was significantly higher in the group of patients in whom we observed a demethylation of the *PD-1* promoter, both compared to patients where no demethylation were observed (48.0% (SD 15.8) vs. 25.7% (SD 9.4) (*p* < .001, 95%-CI [13.1;31.5]), and compared to healthy donors (48.0% (SD 15.8) vs. 24.5% (SD 11.1) (*p* < .0001, 95%-CI [11.7;35.4])) (Figure 5). A trend towards higher *PD-1* baseline methylation in the patients' PBMNCs and T cells compared to those of healthy donors was observed (PBMNCs, *p* = 0.07; 95%-CI [−1.7;38.2], T cells, *p* = 0.16; 95%-CI [−3.0;17.7]).

Comparison of the methylation levels in PBMNCs and T cells from the 10 patients, where data on both cell types were available, reveals a difference in the baseline levels as well as a difference during treatment. In six of the ten patients we observed a good correlation between the relative methylation level in PBMNCs and T cells during treatment, either occurring as demethylation or no demethylation in both cell populations. In one patient we observed a demethylation in PBMNCs and CD4^+^ T cells but not in CD8^+^ T cells, and in three patients demethylation was seen in the compound PBMNC population; however, no demethylation was observed in the T cells at the corresponding time points (Supplementary Figure 1). Accordingly, changes in *PD-1* methylation in PBMNCs do not always reflect the changes in the T cell compartment, emphasizing the importance of analyzing sorted cells.

Altogether, the methylation analyses revealed demethylation of the *PD-1* promoter during 5-aza treatment in 12 of 27 (44%) patients.

### PD-1 expression in CD4^+^ and CD8^+^ T cells from 5-aza treated patients

To investigate whether demethylation of the *PD-1* promoter during 5-aza treatment leads to an increased expression of *PD-1*, we examined the expression of *PD-1* mRNA in the same cells before and during 5-aza treatment. The gene expression was examined in ten patients; *PD-1* promoter demethylation in CD4^+^ and/or CD8^+^ T cells correlated with an increase in *PD-1* expression. No *PD-1* up-regulation was observed in the patients without demethylation of the *PD-1* promoter.

**Figure 6:**
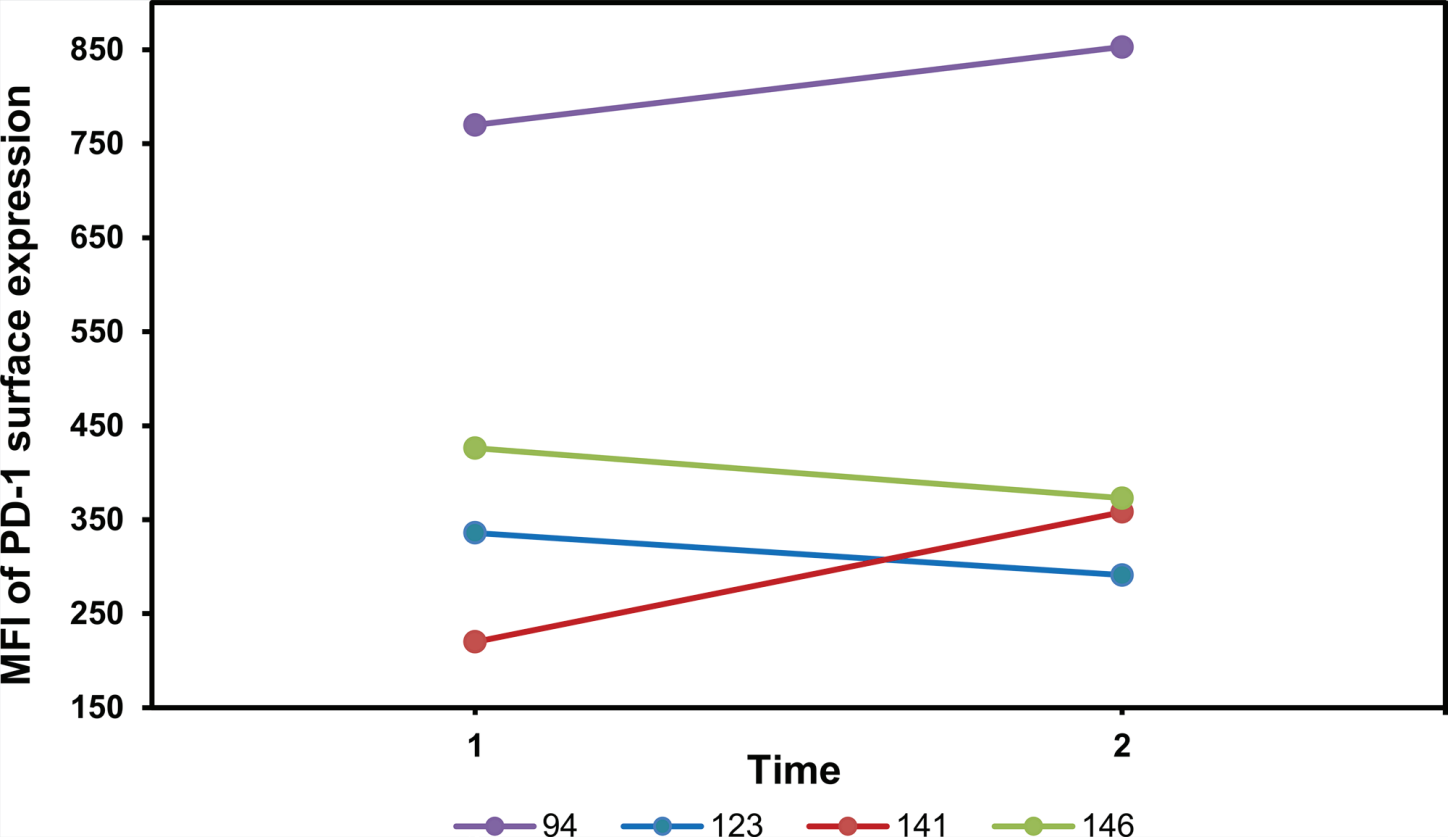
PD-1 protein surface expression on CD8^+^ T cells from 5-aza treated patients *PD-1* protein surface expression, as expressed by the median fluorescence intensity (MFI), on CD8^+^/CD45RO^+^ T cells from PBMNCs of four patients at baseline before 5-aza treatment (1) and following the first treatment cycle (2) (the first available sample for every patient after the first treatment cycle. Patient no. 94, 123, 141 and 146: C4D5, C3D1, C2D1 and C3D1, respectively (C = course of 5-aza treatment. D = day in treatment course)). In patient no. 94 and 141 an increase in PD-1 protein expression is observed (MFI: 770 to 853 (MFIR: 1.11), and MFI: 220 to 358 (MFIR: 1.63), respectively), whereas in patient no. 123 and 146 no increase is observed (MFI: 336 to 291 (MFIR: 0.89), and MFI: 426 to 373 (MFIR: 0.88), respectively). Correspondingly, we observed a significant demethylation of the *PD-*1 promoter during treatment in CD8^+^ T cells in patient no. 94 and 141 (Figure 3B) and no demethylation in patient no. 123 and 146 (Figure 4B). Moreover, we saw a higher baseline MFI of PD-1 in patient 94 compared to patient 141, which is in line with the variation in baseline methylation level.

Moreover, to support the correlation between increased *PD-1* mRNA and cell surface protein expression, we performed multiparameter flow cytometry on PBMNCs from four patients by measuring the PD-1 expression (median fluorescence intensity, MFI) on CD8^+^ T cells expressing the activating marker CD45RO (Figure 6). The two patients showing a *PD-1* promoter demethylation and increase in *PD-1* mRNA expression following 5-aza treatment markedly increased the PD-1 protein surface expression level too, compared to the other two patients with no demethylation of the *PD-1* promoter.

### Correlation between methylation and expression in CD4^+^ and CD8^+^ T cells from 5-aza treated patients

**Figure 7:**
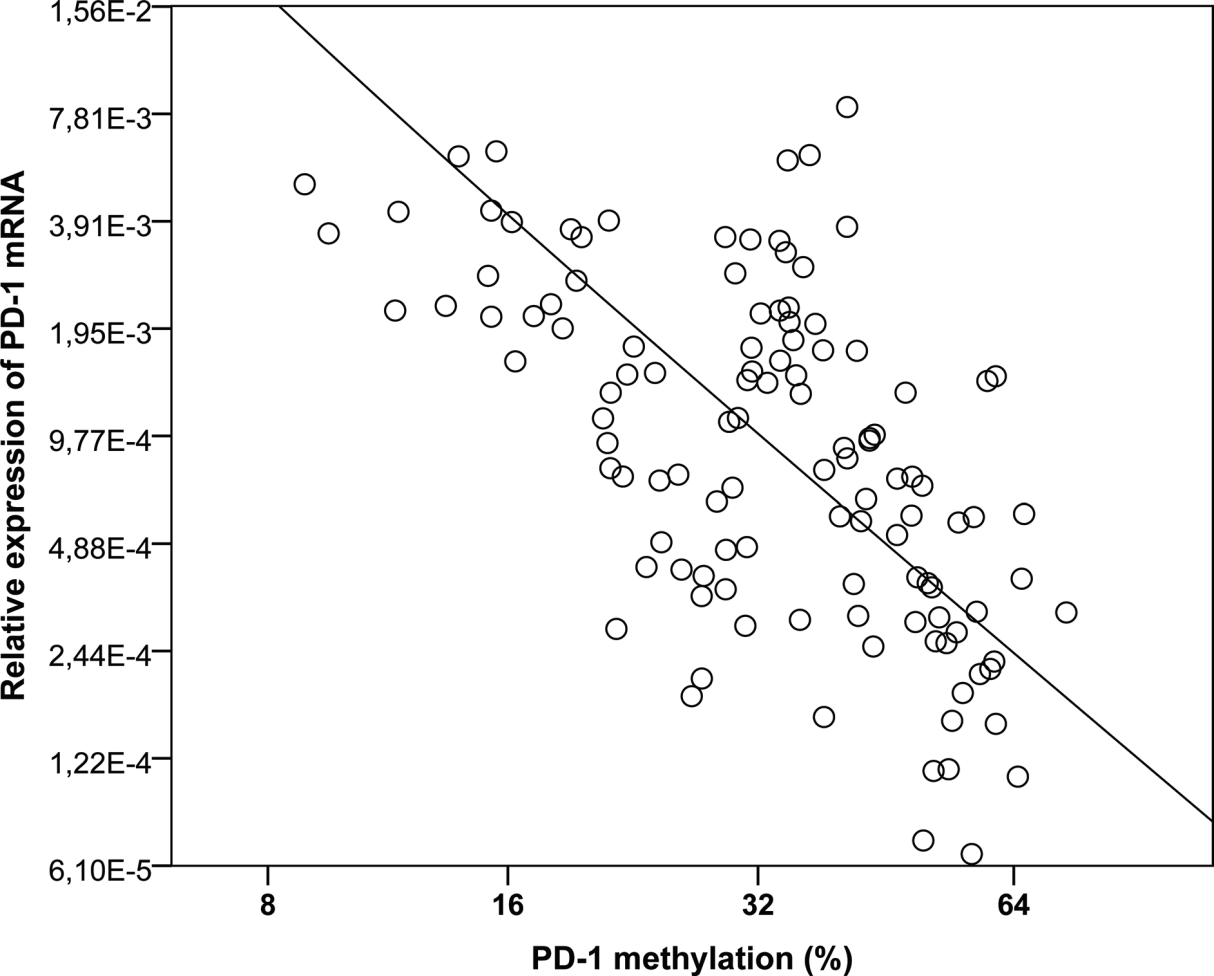
Correlation between *PD-1* promoter methylation and *PD-1* gene expression in peripheral blood CD4^+^ and CD8^+^ T cells from 5-azacytidine treated patients Ten patients and their corresponding values of methylation and relative expression before and during 5-aza treatment (altogether 117 pairs of observations). Methylation level is plotted as co-variate and the relative expression as outcome on a log-log scale with the base 2. The linear regression reveals a statistically significant inverse relationship between the two log-transformed variables (*p* < .0001) with the regression coefficient −2.0936 (SE 0.2521).

Next we compared the *PD-1* gene expression to the *PD-1* promoter methylation levels in T cells from the ten patients where corresponding values of the parameters were available. We found a statistically significant inverse correlation between the *PD-1* promoter methylation and expression level (*p* < 0.0001) (Figure 7).

### Correlation between clinical response and PD-1 promoter methylation

**Figure 8:**
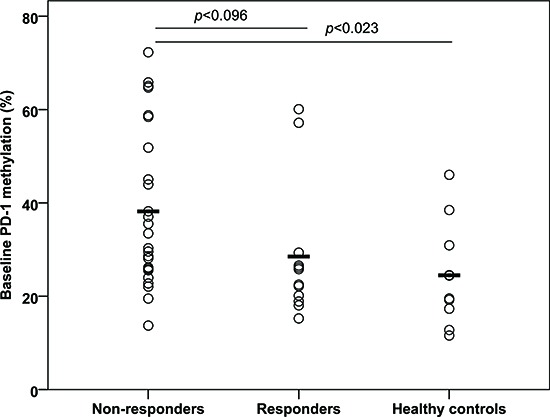
Baseline *PD-1* promoter methylation in peripheral blood CD4^+^ and CD8^+^ T cells from 5-azacytidine treated patients and five healthy donors The patients are grouped according to whether they responded or not responded to the treatment. The values for CD4^+^ and CD8^−^ T cells are pooled.

Due to PD-1′s inhibiting role on anti-tumor immune responses, we wanted to investigate whether demethylation of the *PD-1* promoter in T cells and PBMNCs during 5-aza treatment is correlated to the clinical response. A significant difference in overall response rate (ORR) was observed when comparing patients with and without *PD-1* promoter demethylation (Table 1). In the group of patients with *PD-1* promoter demethylation, only one of 12 (8%) patients showed an overall response to 5-aza. Of the 15 patients with no demethylation an overall response was observed in 9 patients (60%). The ORR was significantly higher in the group of patients without *PD-1* promoter demethylation (*p* = 0.014). Furthermore, a trend towards longer OS was seen in the patients without *PD-1* promoter demethylation (*p* = 0.11) (Supplementary Figure 2). Non-responding patients had a significantly higher baseline PD-1 promoter methylation compared to healthy controls (38.2% vs. 24.5%; *p* = 0.023, 95%-CI [2.0;25.4]) and a trend towards a higher baseline PD-1 promoter methylation compared to responding patients (28.5% vs. 38.2%; *p* = 0.096, 95%-CI [−21.1;1.8]) (Figure 8). Taken together, these data indicate that demethylation of the *PD-1* promoter in T cells during 5-aza treatment might be associated with a poorer clinical response to 5-aza.

## DISCUSSION

Treatment with HMAs has become the standard of care in higher-risk MDS patients who are not eligible for allogeneic hematopoietic stem cell transplantation, and is also approved for CMML and AML patients with 20–30% blasts. Even though the effects of single agent therapy with HMAs are encouraging, remissions are not long lasting, and novel treatment modalities are urgently needed in MDS. Within recent years immune modulating agents have shown promise as novel anti-cancer agents in both experimental and clinical studies in solid tumors [24, 25].

It was recently shown that up-regulation of the immune inhibitory receptor PD-1 on T cells during infections is coupled to demethylation of CpG sites in the *PD-1* promoter [18]. During the development of T cells from naïve to activated and memory T cells, the *PD-1* promoter changes from methylated to unmethylated and back to methylated again. Under normal circumstances, demethylation of the *PD-1* promoter appears to be mediated by ligation of the T cell receptor (TCR) [26]. Youngblood et al. showed that 5-aza was able to sustain a high expression of *PD-1* mRNA and protein *in vitro*, indicating that DNA methylation causes PD-1 repression. Interestingly, during chronic infections, the *PD-1* promoter remains unmethylated with continuous PD-1 overexpression in the chronically activated T cells, leading to exhausted and dysfunctional T cells [27]. Furthermore, it appears that persistent antigen expression by malignant tumors, like chronic infections, can promote the expression of inhibitory surface molecules on T cells, which can cause functional T cell exhaustion [28]. Our study suggests that 5-aza treatment may facilitate the exhaustion of tumor-specific T cells in patients and provides a possible explanation for the development of 5-aza resistance.

One recent study by Yang et al. has examined the effect of HMAs on *PD-1* expression and methylation during the first treatment cycle. In a heterogeneous cohort of 61 patients (MDS, CMML and AML) treated with HMAs (the majority (87%) in combination with other agents) up-regulation (≥2-fold) of *PD-1* gene expression in PBMNCs was observed during the first cycle of therapy in 58% of the patients. In a subgroup of 18 patients *PD-1* promoter methylation in PBMNCs was investigated, and the baseline methylation levels were higher in resistant patients as compared to responding patients – a trend also observed in the current study of sorted T cells. Moreover, Yang et al. observed that the methylation level during the first treatment course was more dynamic in resistant patients as compared to responding patients [22].

Here, we have demonstrated that *in vivo* treatment with 5-aza has direct impact on the expression of *PD-1*. Treatment with 5-aza was accompanied by a loss of DNA methylation in the *PD-1* promoter in 44% (12 of 27) of the patients and the methylation level of the promoter was inversely correlated with *PD-1* gene expression in T cells. Flow cytometry analyses of the PD-1 protein expression on CD8^+^ T cells supported the correlation between the increase in *PD-1* mRNA and the expression of PD-1 cell surface protein. Our combined analyses of both PBMNCs and sorted T cells in ten patients revealed that the *PD-1* promoter methylation changes observed in the PBMNCs do not always reflect the methylation changes in the T cell population. This is probably due to changes in the composition of the PBMNCs rather than changes in the *PD-1* promoter methylation, emphasizing the importance of sorting out the relevant cells in such studies.

Interestingly, we found a significantly higher ORR and a trend towards a better OS in patients without demethylation of the *PD-1* promoter during 5-aza treatment. The baseline *PD-1* methylation level was significantly higher in patients where a demethylation was observed, both compared to normal controls and to patients where no demethylation was observed. Furthermore, a significantly higher baseline *PD-1* methylation level was observed in non-responding patients compared to healthy controls. In addition, we demonstrate that a clinical relevant *PD-1* demethylation is not limited to the first treatment cycle but occurs throughout the treatment course. Accordingly, we believe that our study, on isolated T cell populations and with longer follow-up time, is an important extension to the initial findings of Yang et al.

A few studies indicate that PD-L1 and PD-L2 is overexpressed in higher-risk MDS [22, 23] and that PD-L1 may also be induced by 5-aza treatment [29]. It has furthermore been shown that expression of PD-L1 is an independent negative prognostic factor in different malignancies [13, 15]. This relates to the fact that the PD-1/PD-L1 pathway is a central mediator of T cell exhaustion (as well as inducer of T cell apoptosis [17]). Thus, expression of the PD-1 ligands on tumor cells appears to be an important way of evading the specific tumor immune response. Recent clinical trials with blockade of the PD-1 pathway have shown significant clinical responses in solid tumors [30, 31]. The flow cytometry data presented in this study together with previous *in vitro* and murine studies [18, 23, 32] indicate a positive correlation between *PD-1* gene expression and surface expression *in vivo*; thus, we believe that the observed *PD-1* demethylation and mRNA up-regulation lead to increased PD-1 surface expression on activated T cells.

Interestingly, we show that demethylation of the *PD-1* promoter during 5-aza treatment correlates with a poorer ORR. This suggests that *PD-1* up-regulation in T cells caused by 5-aza might be involved in the impaired response/development of resistance to 5-aza treatment observed in a substantial fraction of patients. Obviously, our findings need validation in a larger and uniformly treated cohort. However, this study provides an important demonstration of 5-aza being able to up-regulate *PD-1*, a key immunoinhibitory receptor, in patients' T cells *in vivo*. We believe that the observed effect of 5-aza on *PD-1* may accelerate the development of dysfunctional effector T cells leading to primary or secondary resistance. We therefore suggest that combination therapy with a HMA and a PD-1 pathway inhibitor in the treatment of higher-risk MDS will release this “brake” on the tumor-specific T cells, potentially enhancing their function. A reinforced immune response against the malignant blasts may improve the clinical outcomes in MDS.

## MATERIALS AND METHODS

### Patients and treatment

Peripheral blood (PB) was sampled from 27 patients with MDS (*n* = 19), CMML (*n* = 3) and AML (*n* = 5) and 5 healthy donors. The patient samples were collected at the Department of Hematology, Rigshospitalet, Copenhagen, and Aarhus University Hospital, Aarhus, between 2008 and 2011. The study was approved by the Regional Ethical Committee. All patients had signed informed consent according to the Declaration of Helsinki. All patients were diagnosed according to the World Health Organization (WHO) criteria [33], and the International Prognostic Scoring System (IPSS) [34] was used to stratify the MDS patients into risk-groups. Seven patients were classified as IPSS intermediate-1; they all progressed before the initiation of 5-aza treatment. The patients were treated with 100 mg/m^2^ s.c. 5-aza (Vidaza, Celgene, NJ) for five consecutive days in each 28-day cycle, according to the Nordic MDS guidelines (http://www.nmds.org/Nordic-Care-Programme). PB samples were collected from each patient before 5-aza administration on day one and day five in each individual course of 5-aza treatment.

The clinical response to treatment was evaluated in accordance with the revised International Working Group (IWG) response criteria [35]. Overall response was defined as complete remission (CR), partial remission (PR), marrow CR with hematologic improvement (HI) and stable disease (SD) with HI. CR in the bone marrow without HI and SD without HI were defined as no response.

Patient characteristics and response to treatment are summarized in Table 1.

### Isolation of mononuclear cells and sorting of CD4^+^ and CD8^+^ T cells

Peripheral blood mononuclear cells (PBMNCs) were isolated from peripheral blood immediately after sampling by using Ficoll-Paque PLUS (GE Healthcare) density-gradient centrifugation and thereafter cryopreserved at ~−196°C for later use. The frozen PBMNCs were thawed and thereafter immediately separated into CD4^+^ and CD8^+^ T cells using magnetic bead-based separation on a RoboSep device according to the manufacturer's instructions (StemCell Technologies). Separation of cell subsets from the healthy donors was performed on freshly isolated PBMNCs.

### Nucleic acid extraction

Genomic DNA from MNCs was isolated using Gentra Puregene Cell Kit (Qiagen). Genomic DNA and total RNA from separated CD4^+^ and CD8^+^ T cells were extracted using the AllPrep DNA/RNA/miRNA Universal Kit (Qiagen) according to the manufacturer's protocol. The DNA and RNA quantity and quality were controlled on a spectrophotometer (Eppendorf BioPhotometer).

### Bisulfite conversion and pyrosequencing

250 ng of genomic DNA was bisulfite converted using the EZ DNA Methylation kit (Zymo Research) with a slight modification. Samples were incubated at 42°C for 30 minutes instead of 37°C for 15 minutes. For the bisulfite reaction the alternative incubation conditions described in the appendix were used. The bisulfite-treated DNA was amplified by performing polymerase chain reaction (PCR) with locus-specific primers for the *PD-1* gene promoter using PyroMark Gold master mix. Forward primer: 5′-TTTGTGGATGGTTTTATATTATGGTTATAG-3′. Reverse primer: 5′-biotin-TCACAACCAACCCCTACC-3′. The PCR was performed on the Gene PCR System 9700 (Applied Biosystems). PCR cycling conditions were as follows: One cycle of 95°C for 15 min, followed by 45 cycles of 94°C for 20 s, 60°C for 20 s and 72°C for 20 s, and one cycle of 72°C for 10 min. Methylation analysis was done by pyrosequencing of the *PD-1* promoter carried out on a PyroMark Q24 (Qiagen) using the PyroMark Gold Q24 reagents (Qiagen), according to the manufacturer's instructions. Sequencing primer: 5′-ATTATGGTTATAGTTTTAGATTTTT-3′. The design of the pyrosequencing assay was based on a former publication [18]. The assay covers three CpG-sites in the upstream DNA-sequence of the transcriptional start site of the *PD-1* gene (Supplementary Figure 3) [18]. The *PD-1* methylation level for a given sample was calculated as a mean of the methylation % of the three investigated CpG-sites.

### Real-time quantitative polymerase chain reaction

250 ng of total RNA was used for reverse transcription reactions to generate cDNA using SuperScript III Reverse Transcriptase and random hexamer primers (Invitrogen). Real-time quantitative PCR (RT-qPCR) of the *PD-1* cDNA was performed using TaqMan assays (id. Hs01550088_m1, Applied Biosystems) [36]. The amplification was performed on a LightCycler 480 II instrument. The amplification curves were analyzed using Roche LightCycler version 1.5.1.62 software for determination of crossing point (Cp, by the second derivative method). All RT-qPCR assays were carried out in duplicate and then repeated with new cDNA synthesis. Reverse transcriptase negative cDNA synthesis reactions were performed for at least one sample per plate. Gene expression was normalized to endogenous *GAPDH* (TaqMan assay, id. Hs99999905_m1) expression and relative gene expression was calculated by using the comparative threshold method (2^−ΔΔ*C*T^ method [37]) normalized to cDNA from T cells from the same patient at baseline of 5-aza treatment.

### Flow cytometry

Cryopreserved PBMNCs were thawed, incubated with an Fc receptor blocking reagent (ChromPure Mouse IgG, Jackson ImmunoResearch Suffolk, UK) to inhibit non-specific binding before incubated with the following, pre-titrated antibodies (all from BD Biosciences, San Jose, CA): anti-CD3 V450, anti-CD45RO FITC, anti-PD-1 (CD279) PE, and anti-CD8 AF700. Flow cytometry was acquired by a LSRFortessa (BD Biosciences, San Diego, CA) and data analyzed using FlowJo version 10.2 (Tree Star Inc., Ashland, OR). Compensation was performed using BD CompBeads (BD Biosciences). Applying gating strategies based on unstained controls and for PD-1 the fluorescence minus one (FMO), the expression of PD-1 was measured on CD3^+^/CD45RO^+^/CD8^+^ singlets and given by the median fluorescense intensity (MFI) (Supplementary Figure 4).

### Statistical analysis

Differences in clinical characteristics for patients with or without demethylation of the *PD-1* promoter during 5-aza treatment were compared using the Pearson chi-squared test, or Fischer's exact test if appropriate, for categorical variables and the Wilcoxon rank sum test for continuous variables. Overall survival was analyzed by the Kaplan-Meier method and compared using the log-rank test. Survival was measured from the onset of 5-aza therapy. Patients who were alive were censored at the date of last follow-up. Patients who underwent BM transplantation were censored at the day of transplantation. Statistically significant changes in gene promoter methylation were assessed by a linear mixed model (a three-level model) because of the correlation of within-patient measurements. Regression analysis of the association of gene promoter methylation and gene expression was calculated using mixed models due to the occurrence of repeated measurements. The mixed model analyses were performed using the SAS Enterprise Guide 4.3 (SAS Institute, NC). All other statistical analyses were performed using SPSS version 22.0.0 (IBM, NY). Significance level was 5% and two-sided for all analyses.

## SUPPLEMENTARY FIGURES


