# Supplementary material for: Hypomethylation and up-regulation of PD-1 in T cells by azacytidine in MDS/AML patients: A rationale for combined targeting of PD-1 and DNA methylation
Source: Oncotarget. 2015 Mar 18;6(11):9612–26. doi: 10.18632/oncotarget.3324 (PMC4496243; doi:10.18632/oncotarget.3324)
Supplement: Supplementary file 1 [file oncotarget-06-9612-s001.pdf]

SUPPLEMENTARY FIGURES

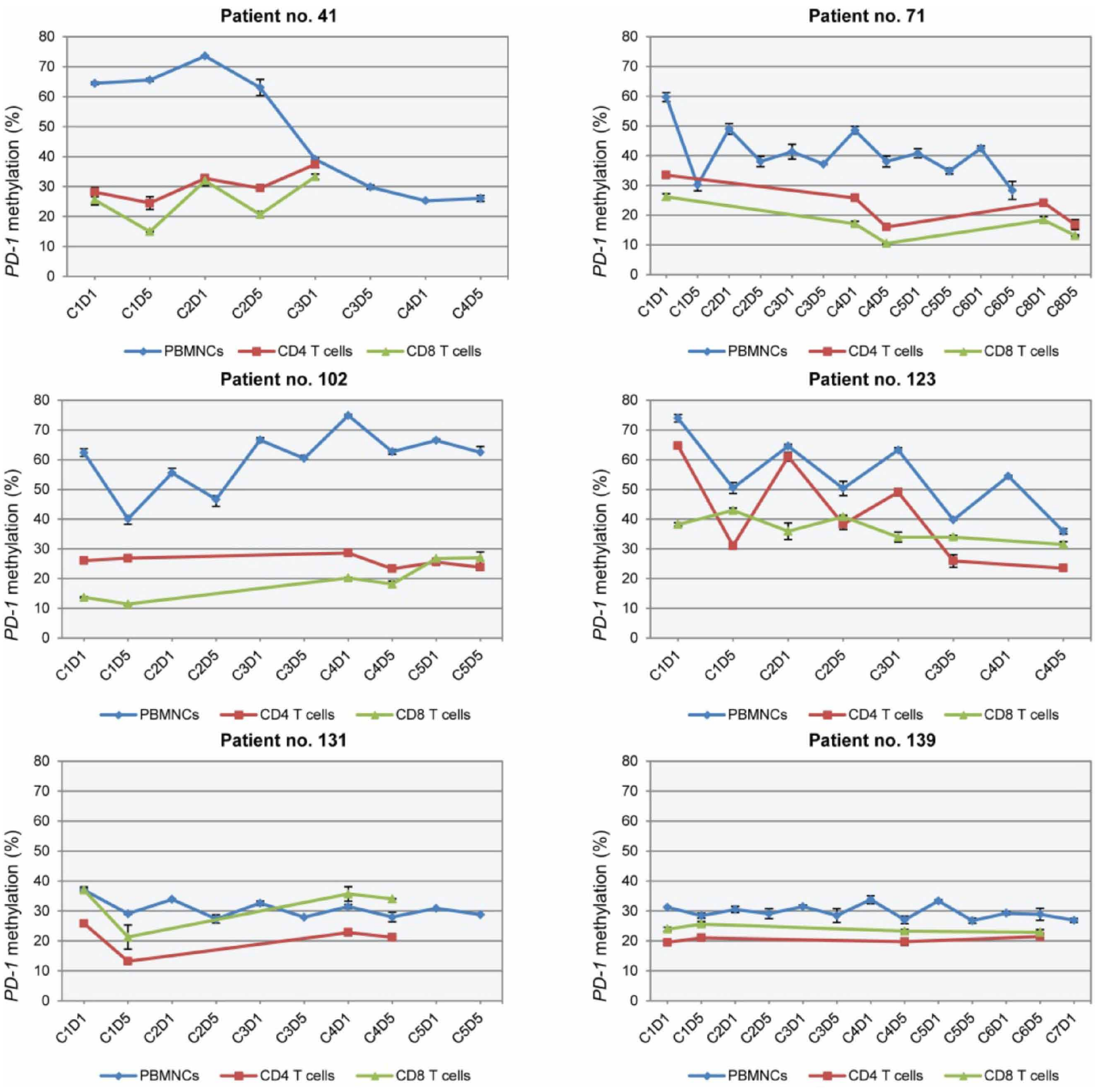

Supplementary Figure S1: Dynamics of *PD-1* promoter methylation in peripheral blood mononuclear cells and CD4$^+$ and CD8$^+$ T cells from 10 patients during treatment with 5-azacytidine. In six of the ten patients (patient no. 71, 131, 139, 141, 146 and 158) we observed a good correlation between the relative methylation level in PBMNCs and T cells during treatment, either occurring as demethylation or no demethylation in both cell populations. From patient no. 41 material was limited, and CD4$^+$ and CD8$^+$ T cells were sorted until day one in the third treatment course. In the first course we observed demethylation in the CD8$^+$ T cells, but not in the CD4$^+$ T cells and PBMNCs. (*Continued*)

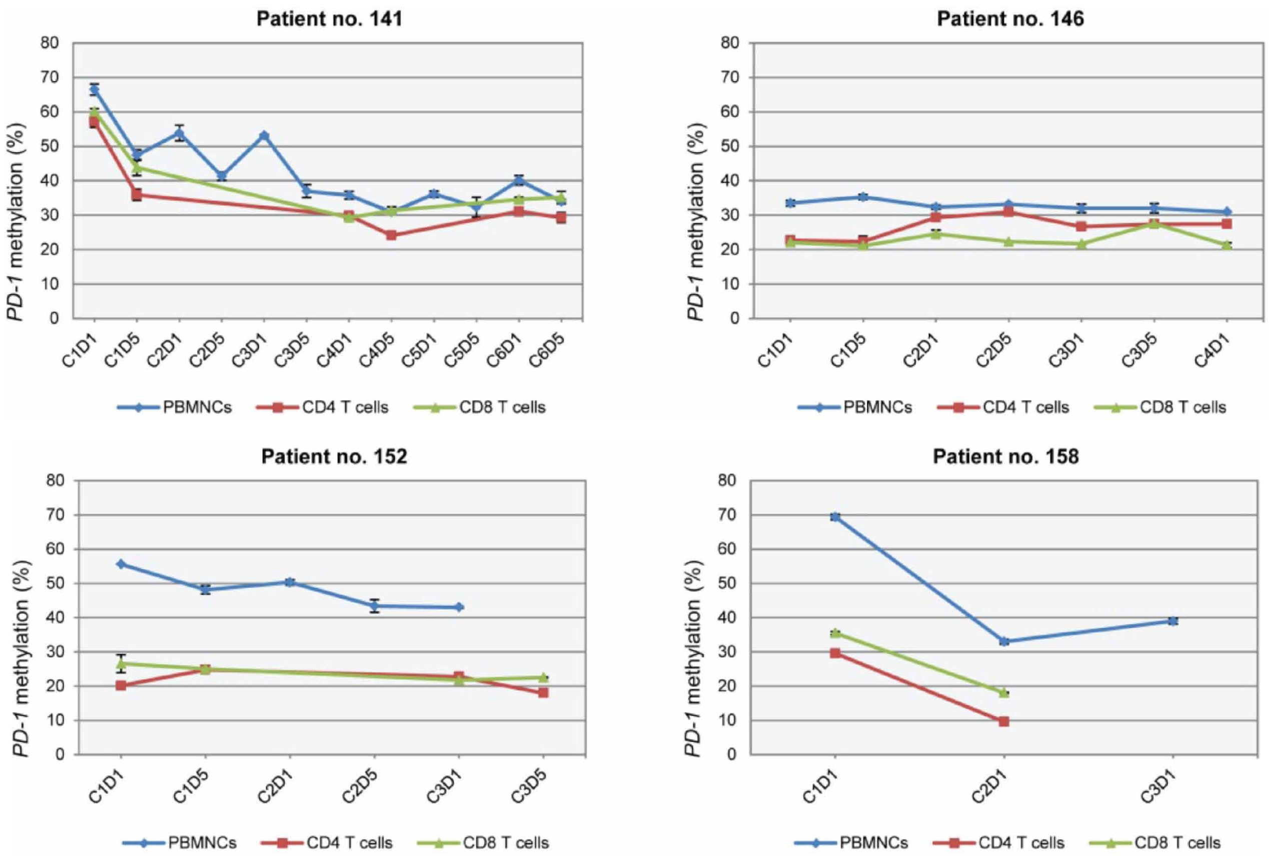

Supplementary Figure S1: (*Continued*) Demethylation was observed from the third course and onwards in the PBMNCs. In patient no. 102 and 152 we observed demethylation in the PBMNCs; however, no demethylation was observed in the T cells in the corresponding courses. Finally, in one patient (patient no. 123) we observed demethylation in PBMNCs and $CD4^{+}$ T cells but not in $CD8^{+}$ T cells. C = course of 5-aza treatment. D = day in treatment course.

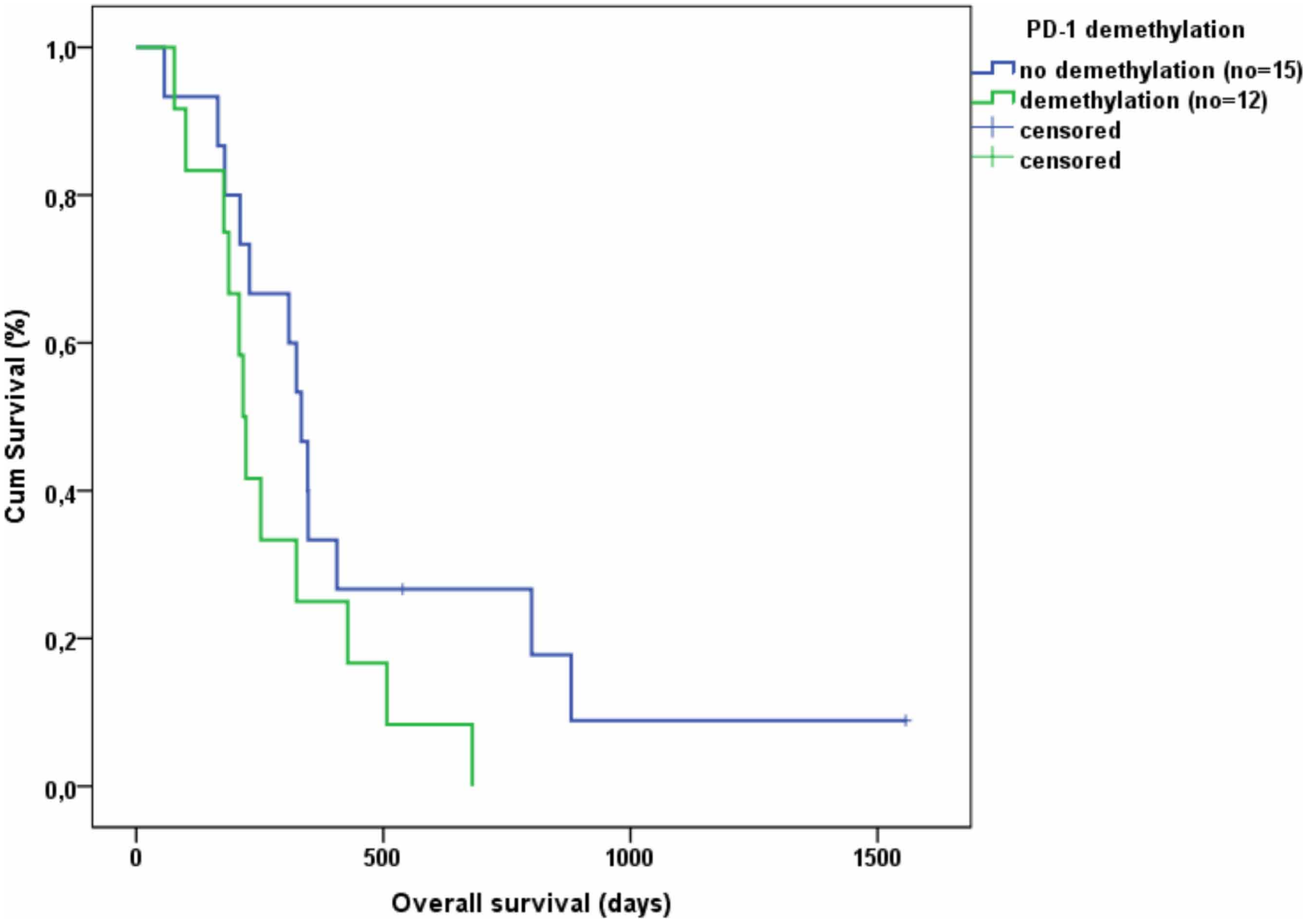

Supplementary Figure S2: Kaplan-Meier estimates of overall survival and *PD-1* promoter demethylation in T cells and peripheral blood mononuclear cells from 5-azacytidine treated patients. The curves represent the overall survival among patients with a classifiable *PD-1* demethylation (12 patients) compared with patients with no *PD-1* demethylation (15 patients) during 5-azacytidine treatment in peripheral blood $CD4^+$ and $CD8^+$ T cells and mononuclear cells (see Table 1). Each tick mark represents a censored patient. There was a trend towards a better overall survival among patients without *PD-1* demethylation compared to patients with *PD-1* demethylation; median OS times were 334 months and 216 months, respectively ($p = 0.11$ by log-rank test).

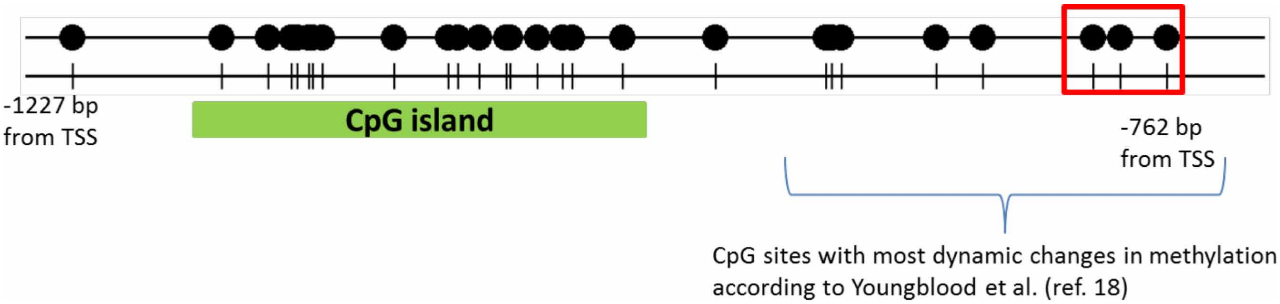

Supplementary Figure S3: CpG-sites in Conserved Region C of the *PD-1* gene promoter. The red box indicates the three CpG-sites investigated in this study. TSS = transcription start site. Bp=base pairs.

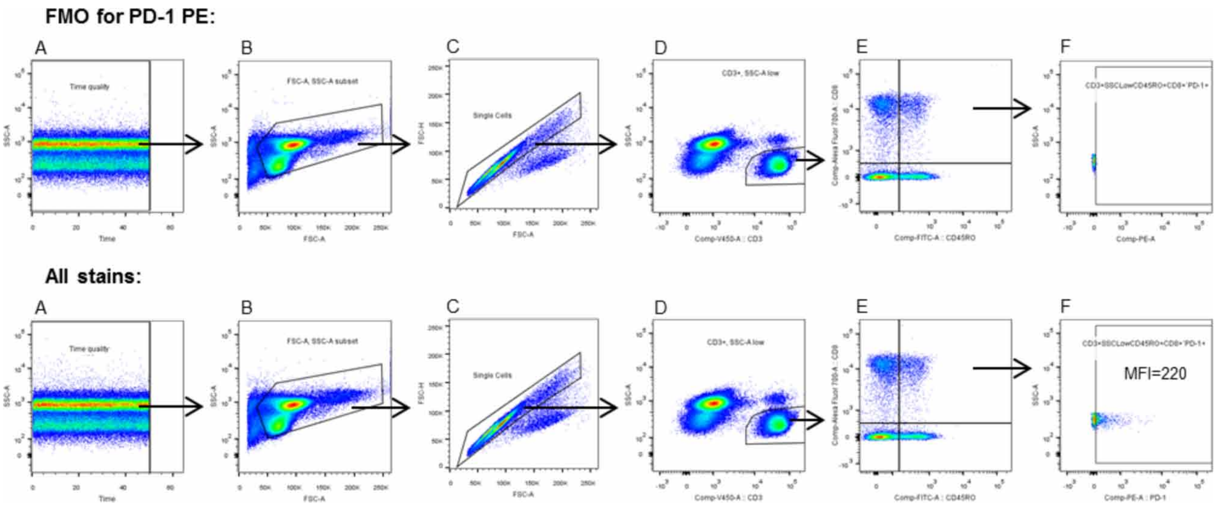

Supplementary Figure S4: Representative flow cytometric data and gating strategy. The baseline sample for patient no. 141 is shown (the fluorescence minus one (FMO) for *PD-1* PE and all stains). A sequential and stringent gating strategy was used in all analyses including an initial time quality control **(A)**. Mononuclear cells were gated on a forward scatter/side scatter (FSC/SSC) plot excluding debris (FSC^{low}/SSC^{low}) **(B)** followed by singlet gating (FSC-A/FSC-H) **(C)**. T cells were identified as $CD3^+/SSC^{med}$ **(D)** and the activated/primed $CD8^+$ T cell subset identified as $CD3^+/CD45RO^+/CD8^+$ **(E)** were analyzed for the expression of PD-1 **(F)** given by the median fluorescense intensity (MFI).
